# Retrospective evaluation of inpatients admitted to a tertiary hospital in Somalia for Pediatric surgery

**DOI:** 10.4314/ahs.v22i1.80

**Published:** 2022-03

**Authors:** Yeliz Kart, Cüneyt Uğur, Abdishakur Mohamed Abdi

**Affiliations:** 1 Department of Pediatric Surgery, University of Süleyman Demirel, Faculty of Medicine, Isparta, Turkey. MD. yelizkart@yahoo.com; 2 Department of Pediatrics, University of Health Sciences Turkey, Konya Health Application and Research Center, Konya, Turkey. MD. cugur70@gmail.com; 3 Department of Pediatric Surgery, Recep Tayyip Erdogan Training and Research Hospital, Mogadishu, Somalia. MD. howraarta@gmail.com

**Keywords:** Surgery, children, epidemiology, mortality, Somalia

## Abstract

**Objective:**

The aim is to reflect on the epidemiology of the patient population at a tertiary hospital for pediatric surgery, diagnostic pattern, and mortality in Somalia retrospectively.

**Methods:**

In this study, 163 patient who were hospitalized to Pediatric Surgery Clinic of Mogadishu Somalia Turkey Recep Tayyip Erdoğan Training and Research Hospital in 2018 were included. Data regarding age, gender, diagnosis, surgical condition, mortality rate and cause of the death were recorded from the patient charts and the institutional digital database

**Results:**

Of 163 patients 47 were female (28.8%) and 116 were male (71.2%). The mean age of the patients was 6.4 ± 4.8 years. The main diagnoses were congenital malformation (34.4%), acute abdomen (25.8%), traumatic injury (23.3%), infection (9.8%) and neoplasm (6.1%). Mortality rate was 9.8% and the leading cause of death was sepsis by 87.5%. Perforated appendicitis, intestinal obstruction and intussusception were creating the 68.7% of the diseases that result in death.

**Conclusions:**

Our results show that two-thirds of the surgical deaths could be prevented with timely presentation. We think that the health policymakers in Somalia should focus on how to improve the access to surgical care, patient transfer, timely presentation, and training of pediatric surgeons and to overcome the poor surgical outcomes.

## Introduction

Somalia is one of the worst in the world concerning the health indicators. The lack of a centralized government since the civil war which occurred in 1991 has led to a profound decline in healthcare service. The country is currently ranked as one of the most dangerous places in the world for mothers to give birth. The infant mortality in Somalia is ranked the fifth highest in the world and the maternal mortality is 200 per 100,000 live births by 2008 estimates. Moreover, there is a critical shortage of the health workforce in Somalia. Number of the physicians and the nursing staff was less than 1 per 1000 people in Somalia by 2010^1^. The life expectancy at birth is 55 years, and lower considerably than the neighboring Kenya and Ethiopia^2^.

Somalia has become dependent to the foreign aid since the country was destroyed by the civil war. However, despite the widespread foreign funding, resources for healthcare in a country with extreme poverty still remain unaffordable. The hospitals, medical technology, the health workforce, and research and capacity building are the major non-funded areas of the health sector^3^. A critical issue obstructing the provision of the sufficient funding is probably the lack of the information regarding the health care facilities and the health workforce. Despite the high mortality in the pediatric population, data concerning the healthcare facilities for this population is also lacking.

The aim was to reflect on the epidemiology of the patient population at a tertiary hospital for pediatric surgery, diagnostic pattern, and mortality in Somalia retrospectively. In the literature, we did not find data about pediatric surgical diseases in Somalia. Thus, we hope that results of this study will provide valuable insight into the surgical conditions that affect children living in Somalia.

## Methods

This study was conducted in Mogadishu Somalia Turkey Recep Tayyip Erdoğan Training and Research Hospital retrospectively. In this study, 163 patient who were hospitalized to Pediatric Surgery Clinic of the hospital, in 2018 were included. Data regarding age, gender, diagnosis, surgical conditions, length of hospital stay, mortality rate and cause of the mortality were recorded from the patient charts and the institutional digital database. Mogadishu population is around 3 million. There are a total of 10 major hospitals and around 40 ambulances in Mogadishu. Hospitals and ambulances are chargeable. Most of the patients come to the hospital with their own means. Ambulance only is used for major casualties such as explosions and traffic accidents This study was approved by the Institutional Ethical Committee and was performed in accordance with the recent version of the Helsinki Declaration (MSTH-5546-611).

The frequency of the pediatric surgical conditions and the mortality rate from surgery were the primary outcome measures of this study.

Descriptive statistics were used to compare the general characteristics of all participants. Test of Normality, including Kolmogorov-Smirnov and Shapiro-Wilk tests, was used to determine the distribution of data. The data were given as mean ± standard deviation (minumum-maximum). Categorical variables were shown as number (n) and percentage (%). Statistical Package for Social Sciences (SPSS) Windows software (ver. 22; IBM SPSS, Chicago, USA) was used for all statistical analyses.

## Results

In this study, we recorded 163 patients. Of patients 47 were female (28.8%) and 116 were male (71.2%). The mean age of the patients was 6.4 ± 4.8 years (1 months-17 year). The age range of the patients was < 1 year (12.9%), 1–4 years (30.1%), 5–9 years (27.6%) and 10–17 years (29.4%). The main diagnoses were congenital malformation (34.4%), acute abdomen (25.8%), traumatic injury (23.3%), infection (9.8%) and neoplasm (6.7%). Table 1 shows distribution of main diagnoses by age range.

**Table 1:** Distribution of main diagnoses by age range

	Age range				
	<1 year	1–4 years	5–9 years	10–17 years	Total
	
Main diagnoses	n (%)	n (%)	n (%)	n (%)	n (%)
Acute abdomen	5 (23,8)	7 (14.3)	15 (33.3)	15 (31.3)	42 (25.8)
Infection	0 (0.0)	8 (16.3)	2 (4.4)	6 (12.5)	16 (9.8)
Congenital malformation	8 (38.1)	26 (53.1)	8 (17.8)	14 (29.2)	56 (34.4)
Traumatic injury	6 (28.6)	6 (12.2)	16 (35.6)	10 (20.8)	38 (23.3)
Neoplasm	2 (9.5)	2 (4.1)	4 (8.9)	3 (6.3)	11 (6.7)

Total	21	49	45	48	163

Pediatric surgery patients were hospitalized to the service at most in summer (34.4%), at least in autumn (17.2%). Acute abdomen with 38.1% rate, infection with 37.6% rate, trauma with 44.7% rate, and neoplasm with 36.4% rate were most common in summer. Congenital malformation with 32.1% rate was most common in spring. Table 2 shows distribution of patient numbers and main diagnoses by months.

**Table 2:** Distribution of the patient numbers and main diagnoses by months

	Main diagnoses					
	Acute abdomen	Infection	Congenital malformation	Traumatic injury	Neoplasm	Total
	
Months	n (%)	n (%)	n (%)	n (%)	n (%)	n (%)
Jenuary	3 (7.1)	0 (0.0)	8 (14.3)	0 (0.0)	1 (9.1)	12 (7.4)
February	2 (4.8)	1 (6.3)	3 (5.4)	3 (7.9)	1 (9.1)	10 (6.1)
March	5 (11.9)	3 (18.8)	6 (10.7)	3 (7.9)	1 (9.1)	18 (11.0)
April	5 (11.9)	1 (6.3)	7 (12.5)	1 (2.6)	1 (9.1)	15 (9.2)
May	4 (9.5)	1 (6.3)	5 (8.9)	2 (5.3)	1 (9.1)	13 (8.0)
June	8 (19.0)	2 (12.5)	3 (5.4)	4 (10.5)	2 (18.2)	19 (11.7)
July	2 (4.8)	1 (6.3)	5 (8.9)	9 (23.7)	1 (9.1)	18 (11.0)
August	6 (14.3)	3 (18.8)	5 (8.9)	4 (10.5)	1 (9.1)	19 (11.7)
Semtember	2 (4.8)	0 (0.0)	2 (3.6)	2 (5.3)	0 (0.0)	6 (3.7)
October	2 (4.8)	1 (6.3)	7 (12.5)	3 (7.9)	1 (9.1)	14 (8.6)
November	2 (4.8)	1 (6.3)	2 (3.6)	3 (7.9)	0 (0.0)	8 (4.9)
December	1 (2.4)	2 (12.5)	3 (5.4)	4 (10.5)	1 (9.1)	11 (6.7)

Total	42	16	56	38	11	163

The three most common congenital malformations were inguinal hernia (6.7%), hypospadias (6.7%) and hydrocele (5.5%). Table 3 shows distribution of congenital malformations. The three most common acute abdomen causes were perforated appendicitis (9.2%), intussusception (6.7%) and intestinal obstruction (4.3%).

**Table 3:** Distribution of congenital malformations

	n	%
Inguinal hernia	11	6.7
Hypospadias	11	6.7
Hydrocele	9	5.5
Hirschsprung's disease	6	3.7
Pyloric stenosis	5	3.1
Undescended testis	4	2.5
Umbilical hernia	3	1.8
Hydronephrosis	3	1.8
Anal atresia	2	1.2
Diaphragmatic hernia	2	1.2

Total	56	34.4

Table 4 shows distribution of acute abdomen causes. The three most common traumatic injury causes were flame burn (8.0%), hot water burn (4.3%) and gunshot wound (3.7%). Table 5 shows distribution of traumatic injury causes. İnfections were abscess (4.9%, n=8), pleurisy (3.7%, n=6) and fournier gangrene (1.2%, n=2). Neoplasms were wilms tumour (2.5%, n=4), lymphangioma (1.2%, n=2), dermoid cyst (0.6%, n=1), thyroid teratoma (0.6%, n=1), neuroblastoma (0.6%, n=1), lymphoma (0.6%, n=1) and ovarian teratoma (0.6%, n=1).

**Table 4:** Distribution of acute abdomen causes

	n	%
Perforated appendicitis	15	9.2
Intussusception	11	6.7
Intestinal obstruction	7	4.3
Appendicitis	5	3.1
Rectal prolapse	3	1.8
Volvulus	1	0.6

Total	42	25.8

**Table 5:** Distribution of traumatic injury causes

	n	%
Flame burn	13	8.0
Hot water burn	7	4.3
Gunshot wound	6	3.7
Blunt injury	4	2.5
Car accident	4	2.5
Bomb injury	2	1.2
Hot oil burn	1	0.6
Electricity burn	1	0.6

Total	38	23.3

The most common diagnoses were perforated appendicitis (9.2%), flame burn (8%), hypospadias (6.7%), inguinal hernia (6.7%) and intussusception (6.7%).

The mean length of hospital stay was 14 ± 14.2 days (1–68 days). Overall mortality rate was 9.8% (16/163). The leading cause of death was sepsis by 87.5% (14/16) and others were severe malnutrition (patient with pyloric stenosis) by 6.2% (1/16) and advanced stage neoplasm (patient with neuroblastoma) by 6.2% (1/16). Among the 16 deaths, children aged < 1 year constituted 25% (4/16), children aged 1–4 years 25% (4/16), children aged 5–9 years 43.8% (7/16) and the 10–17 years old 6.3% (1/16). Almost half of the deaths were among the 5–9 years old.

The three most common diseases that result in death were perforated appendicitis (31.3%), intussusception (25%) and intestinal obstruction (12.5%). These three were creating the 68.7% of diseases that result in death. Table 6 shows distribution of diseases that result in death by age range.

**Table 6:** Distribution of diseases that result in death by age range

	Age range				
	<1 year	1–4 years	5–9 years	10–17 years	Total
Diseases that result in death	n (%)	n (%)	n (%)	n (%)	n (%)
Flame burn	1 (25.0)	0 (0.0)	0 (0.0)	0 (0.0)	1 (6.3)
Hirschsprung's disease	1 (25.0)	0 (0.0)	0 (0.0)	0 (0.0)	1 (6.3)
Hot water burn	1 (25.0)	0 (0.0)	0 (0.0)	0 (0.0)	1 (6.3)
Intestinal obstruction	0 (0.0)	0 (0.0)	1 (14.3)	1 (100)	2 (12.5)
Intussusception	0 (0.0)	4 (100)	0 (0.0)	0 (0.0)	4 (25.0)
Neuroblastoma	0 (0.0)	0 (0.0)	1 (14.3)	0 (0.0)	1 (6.3)
Perforated appendicitis	0 (0.0)	0 (0.0)	5 (71.4)	0 (0.0)	5 (31.3)
Pyloric stenosis	1 (25.0)	0 (0.0)	0 (0.0)	0 (0.0)	1 (6.3)
Total	4	4	7	1	16

## Discussion

The present study provides informations concerning the pediatric surgery cases in Somalia. The results of this study provide valuable insight into the surgical conditions that affect children living in Somalia. Our findings indicate that congenital malformations, acute abdominal pathologies and trauma injuries constitute the majority of the subjects requiring pediatric surgery. Perforated appendicitis and flame burn injury were the most common presenting diagnosis in our records. The mortality rate was in 9.8% of the subjects and sepsis was responsible for the vast majority of the deaths. Unfortunately, acute abdominal pathologies, which are theoretically curable with appropriate surgical intervention, were responsible for two-thirds of the deaths.

Despite the competent data regarding the pediatric surgical diseases from the western countries, accurate information derived from the undeveloped countries, particularly from the sub-saharan Africa (SSA) countries, is limited. Thus, we think that our findings will shed light on the barriers obstructing the provision of a satisfactory health service for the children living in Somalia and allow clinicians to gain understanding of the epidemiology of pediatric surgical conditions.

Mortality from pediatric surgical procedures in high-income countries has demonstrated a significant improvement in the last few decades. However, little is known concerning the demand and the success of the pediatric surgery in low-income countries. There are still major gaps in the surgical care of children living in developing countries. Although the children and adolescents represent 50–60% of the population in SSA, surgical care of this population has attracted little attention^4^–^6^. Pediatric surgery is also a field of deficits for many of the SSA countries excluding South Africa. Most of the SSA countries still face the shortage of health workforce including the pediatric surgeons, anesthetists, neonatologists and pediatric nursing staff 7. The available information suggests that surgery patients account for approximately 6–12% of all pediatric admissions in SSA^8^. Nevertheless, shortages of health workers result in poor access to healthcare for most of the population living in this part of the world.

Previous data from the Gambia, a SSA country, revealed that injuries (46.9%), congenital malformations (24.3%), and infections requiring surgery (14.5%). The most common admission diagnoses for the hospitalized patients were burn injuries (18.8%), osteomyelitis (15.4%), and fractures (12.7%)^9^. Two of the most common pediatric surgical conditions in industrialized countries, appendicitis and hypertrophic pyloric stenosis, were extremely rare in Gambia according to that study. A previous report of Mhando et al. from Tanzania demonstrated similar pattern for the most common pediatric surgical conditions to the information derived in Gambia. The three most common pediatric surgical conditions In Tanzania were injuries (41%), congenital malformations (27%), and infections requiring surgery (18%)^6^. Nonetheless, no such data has been reported from the Somalia up to now. The present study is the first to describe the epidemiology of the pediatric surgical disease in Somalia. Our findings are largely consistent with the previous data reported from Gambia and Tanzania. Congenital malformations (34.4%), acute abdomen (25.8%) and traumatic injuries (23.3%) were the three most common pediatric surgical conditions in Somalia. Although previous reports demonstrated less proportion of patients with acute abdominal surgical pathologies, children suffering from perforated appendicitis (9.2%), intussusception (6.7%) and intestinal obstruction (4.3%) were frequent in our study population. This may be somewhat explained by the tradition and culture which influence the attitude of the family to the management of surgical diseases. The traditional and cultural attitudes are extremely expressive on surgical management of the children living in African and are frequently peculiar to the community from which the child is coming.

The overall mortality of the pediatric surgical cases reported by Bickler et al. from Gambia was 5.3% which is lower than the mortality rate recorded in our study^9^. However, in our study, as an area of violence, some of the injuries were due to the gunshot wound, hot water burn and flame burn which display higher mortality and necessitate special care during hospitalization. Consistent with the published data which provided valuable information from different areas of the SSA, the vast majority of the deaths recorded in this study were due to the sepsis which is in close relationship between the lack of the sanitary conditions and the late presentation of the subjects.

Bickler et al. reported that the highest mortality rates were found among children with meningomyelocele (42%) and posterior urethral valves (40%)^9^. In this study, the three most common diseases that result in death were perforated appendicitis (31.3%), intussusception (25%), intestinal obstruction (12.5%). These were creating two-thirds of the surgical deaths. The difference of our results from Bickler et al results is that there are acute abdominal pathologies that can be treated with an appropriate surgical intervention. The main problem in these subjects is likely the late presentation of the subjects to the hospital. Because the hospital where the study is carried out is one of the most adequate hospitals of the region in terms of both personnel and medical equipment. During this period, the only pediatric surgeon of the region was present in this hospital. We think that this delay depends on economic and social reasons and poor health conditions. We think that these deaths can be reduced by improving access to surgical care, patient transfer, timely diagnosis and treatment, and training of pediatric surgeons.

Our findings for the first time provide longitudinal data concerning the pediatric surgical conditions from the ground, a tertiary center in Mogadishu, the capital of Somali. Our results show that half of the surgical deaths could be prevented with timely presentation. This combination of findings provides valuable information for the decision makers in Somali who should focus on how to improve access to surgical care, patient transfer, timely presentation, and training of pediatric surgeons and to overcome the poor surgical outcomes.

## Conclusion

The present study, for the first time, reports data concerning the epidemiology of the pediatric surgical diseases in Somalia. We consider that this preliminary data from one of the poorest areas of the planet will allow the organizations and communities funding the healthcare system in Somalia to better understand the current status of the children health in this country. We think that be required the comprehension and assumption of poor surgical care as a significant public health problem and that good surgical care can improve the children's health, a progress regarding the pediatric surgical care will occur in Somalia. We also believe that further research is required to from the ground to determine how surgical care should be integrated into child health programs.
